# Maintenance of Kidney Metabolic Homeostasis by PPAR Gamma

**DOI:** 10.3390/ijms19072063

**Published:** 2018-07-16

**Authors:** Patricia Corrales, Adriana Izquierdo-Lahuerta, Gema Medina-Gómez

**Affiliations:** 1 Área de Bioquímica y Biología Molecular, Departamento de Ciencias Básicas de la Salud, Facultad de Ciencias de la Salud, Universidad Rey Juan Carlos. Avda. de Atenas s/n. Alcorcón, 28922 Madrid, Spain; patricia.corrales@urjc.es (P.C.); adriana.zquierdo@urjc.es (A.I.-L.); 2 MEMORISM Research Unit of University Rey Juan Carlos-Institute of Biomedical Research “Alberto Sols” (CSIC), 28029 Madrid, Spain

**Keywords:** PPARγ, metabolism, lipid, RAAS, nuclear receptors, kidney

## Abstract

Peroxisome proliferator-activated receptors (PPARs) are a family of nuclear hormone receptors that control the transcription of specific genes by binding to regulatory DNA sequences. Among the three subtypes of PPARs, PPARγ modulates a broad range of physiopathological processes, including lipid metabolism, insulin sensitization, cellular differentiation, and cancer. Although predominantly expressed in adipose tissue, PPARγ expression is also found in different regions of the kidney and, upon activation, can redirect metabolism. Recent studies have highlighted important roles for PPARγ in kidney metabolism, such as lipid and glucose metabolism and renal mineral control. PPARγ is also implicated in the renin-angiotensin-aldosterone system and, consequently, in the control of systemic blood pressure. Accordingly, synthetic agonists of PPARγ have reno-protective effects both in diabetic and nondiabetic patients. This review focuses on the role of PPARγ in renal metabolism as a likely key factor in the maintenance of systemic homeostasis.

## 1. Introduction

The peroxisome proliferator-activated receptors (PPARs) are ligand-activated nuclear hormone receptors that participate in the transactivation or transrepression of networks of target genes, resulting in complex biological effects. PPARs are class 2 receptors that, upon ligand binding, heterodimerize with retinoid X receptors (RXRs) and translocate to the nucleus, whereupon the PPAR:RXR heterodimer binds to the PPAR response element (PPRE) generally in the promoter region of target genes, to control their expression [1,2]. The affinity of PPARs for ligands, and hence their transcriptional response, is determined by the conformational changes induced by ligand binding within a complex pocket with multiple interaction points [3].

There are three known subtypes of PPARs that have distinct physiological roles in energy metabolism in different tissues: PPARα, PPARδ, and PPARγ. Overall, PPARs function as lipid sensors to govern metabolic homeostasis through binding to dietary metabolites: PPARα regulates catabolism, mainly in the liver and the heart, PPARγ regulates anabolism in adipose tissue, and PPARδ is involved in fatty acid transport and oxidation in skeletal muscle. PPARs not only serve critical roles in the control of lipid metabolism, but they are also implicated in the regulation of vascular diseases, cellular differentiation, insulin sensitization, and cancer [4]. In the kidney, PPARα plays an important role in the metabolic control of renal energy homeostasis and is expressed in the proximal tubules and medullary thick ascending limb, with lower expression in glomerular mesangial cells [5]). Likewise, kidney PPARδ has a role in renal metabolic adaptation to fasting and refeeding [6], and is expressed in the renal cortex and medulla [7].

In humans, the *PPARγ* gene is located on chromosome 3 (3p25.2) and contains nine exons spanning more than 100 kb [8]. Four *PPARγ* splice variants(γ1, γ2, γ3, and γ4), generated by alternative splicing and differential promoter usage, are found in human [9]; however, only two protein isoforms (γ1 and γ2) are encoded [10]. *PPARγ* is also present in other animals (rodents, chicken, lizard, *Xenopus*, and Zebrafish) [11,12], although, they do not appear to display significant functional differences. Mutations in *PPARγ* lead to dysfunctional lipid and glucose homeostasis and have been directly related to type 2 diabetes mellitus and obesity [13], familial partial lipodystrophy type 3 (FPLD3) [14], and also cancer [15,16].

*PPARγ* is expressed predominantly in the adipose tissue where, together with the coexpression of *C/EBP alpha* and other proteins involved in lipid and glucose metabolism, serves as a key regulator both of adipocyte differentiation and triglyceride energy stores, and has pleiotropic vascular effects that are independent of its glucose blood-lowering effect, protecting against the progression of hypertension and atherosclerosis [17,18]. Specifically, *PPARγ1* is expressed in many tissues and cell types, including white and brown adipose tissue, skeletal muscle, liver, pancreatic β-cells, macrophages, colon, bone, and placenta. By contrast, *PPARγ2* has a more restricted pattern of expression with significant amounts found only in white and brown adipose tissue under physiological conditions, although it is induced in liver and skeletal muscle in response to overnutrition or genetic obesity. *PPARγ3* mRNA expression appears to be limited to human white adipocytes, but it is also found in a variety of cell lines with different origins, including liver hepatocellular cells (HepG2), human intestinal cells (Caco-2), and cervical cancer (HeLa) cells. *PPARγ4* is expressed in human adipose tissue [19] and mutations in his promotor are associated to FPLD, familial partial lipodystrophy [20].

At the protein level, PPARγ is subject to several post-translational modifications, including glycosylation and phosphorylation, which function to modify its activity [21]. For example, *O*-GlcNAcylation at Thr-84 has been found to reduce PPARγ transcriptional activity in adipocytes cultured in vitro. Moreover, PPARγ2 is phosphorylated at Ser-112 by MAPK in response to different mitogenic growth factors that inhibit fat cell differentiation. The cdk5-dependent phosphorylation of PPARγ at Ser-273 occurs in inflamed obese white adipose tissue and decreases PPARγ activity. By contrast, PKA phosphorylation has been reported to positively affect the activity of PPARγ [22]. Furthermore, CK-II-dependent PPARγ1 phosphorylation at Ser-16 and Ser-21 is necessary for its nuclear translocation [23,24]. Overall, these events illustrate the complex regulation of this nuclear receptor. Interestingly, *PPARγ* expression can be inhibited by miRNAs in specific settings [25], constituting another layer of regulation.

**Figure 1:**
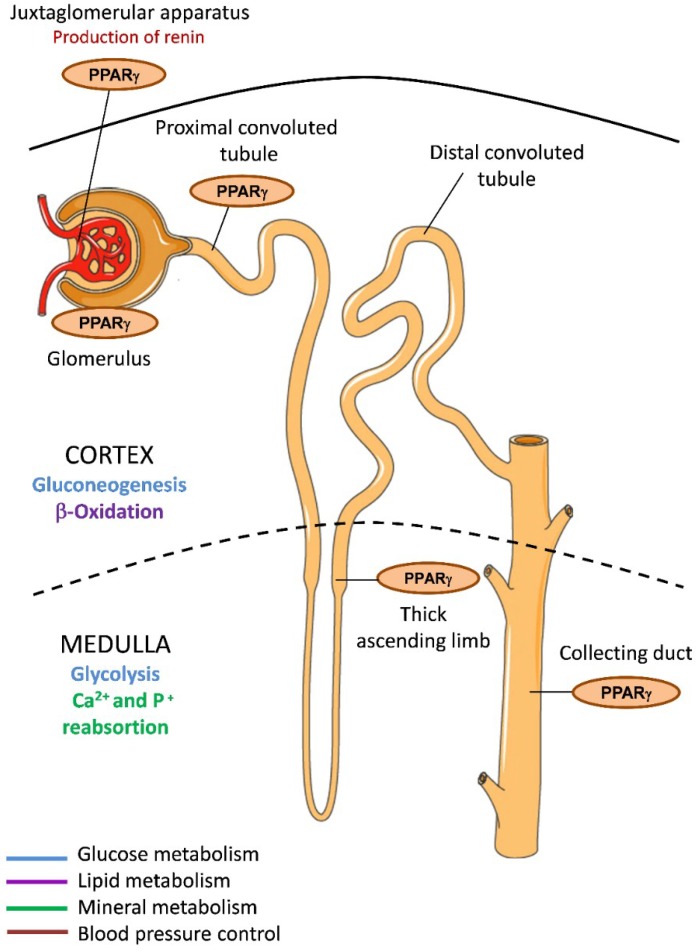
*PPARγ* expression in the kidney and implications for metabolism. PPARγ is expressed in different areas of kidney including the cortex and the medulla, which have different metabolic specializations. Glucose metabolism (blue); Lipid metabolism (purple); Mineral metabolism (green); Blood pressure control (red).

In kidney, PPARγ is expressed in different regions of the renal collecting system under physiological conditions, including connective renal tubules and collecting ducts [26] (Figure 1). PPARγ is also abundant in the inner renal medulla and is localized to the epithelial layer, from the medullary collecting ducts to the urothelium of the ureter and the bladder. It is additionally expressed in renal medullary interstitial cells and in the juxtaglomerular apparatus and the glomeruli, including podocytes, mesangial cells, and renal microvascular endothelial cells [27]. Given that multiple renal cell types have endogenous PPARγ expression and activity, its activation in kidney may be critical for governing renal function. Indeed, as we describe later, synthetic PPARγ ligand agonists have been shown to have reno-protective effects both in diabetic and nondiabetic patients [28].

In this review, we focus on several key observations that illustrate the central role of PPARγ in renal metabolism and maintenance of systemic homeostasis. We will examine the involvement of PPARγ in renal lipid, glucose, and mineral metabolism, and also blood pressure control. Against this background, we will also address the potential use of PPARγ agonists in the clinical setting as therapeutic agents for renal pathologies.

## 2. PPARγ in Renal Lipid Metabolism

Human kidney contains about 3% fat, although this varies greatly among individuals. Much of this (~50%) is in the form of phospholipids that form cell membranes, 15% is in the form of triglycerides, and around 0.3% is nonesterified (free) fatty acids (FFAs) [29]. Under physiological conditions, the kidney can metabolize a variety of substrates, including FFAs, lactate, glutamine, 3-hydroxybutyrate, citrate, pyruvate, α-ketoglutarate, glycerol, proline, and other amino acids. Proximal tubule reabsorption is responsible for about 70% of these substrates, the metabolic fate of which depends on the extracellular medium, hormonal influences, and metabolic conditions (acid-base). Once reabsorbed, FFAs are metabolized within mitochondria of proximal tubule cells by β-oxidation (as their glycolytic capacity is poor), constituting the largest source of Adenosine triphosphate (ATP). An increase in the availability of intracellular fatty acids for mitochondria results in competition with other oxidizable substrates, triggering a decrease in the use of glutamine, with the consequent reduction in ammoniagenesis [30].

Moorhead et al. in 1982 were the first to establish a link between alterations in lipid metabolism and kidney disease [31]. Mutations in enzymes responsible for lipid metabolism or an increase in serum lipids promotes a decline in renal function [32]. Accordingly, mice deficient for PPARγ present alterations in renal lipid metabolism, and the extent of renal damage induced by a high-fat diet is lower in PPARγ heterozygous knockout mice than in wild-type mice concomitant with a decrease in lipid accumulation and lipogenesis and an attenuation of lipid-mediated kidney damage [33]. We recently demonstrated that accelerated kidney damage manifests in the POKO mouse—A model of the metabolic syndrome generated by ablation of the *PPARγ2* isoform in a leptin-deficient obese (*ob*/*ob*) background [34,35]. Despite having similar body weight and blood pressure to *ob*/*ob* littermates at an early age, POKO mice present renal hypertrophy and dyslipidemia, and with alterations in some proliferation markers. Moreover, they develop incipient insulin resistance associated with a decrease in the expression of renal adiponectin. POKO mice also exhibit faster progression of kidney disease compared with *ob*/*ob* mice, accompanied by an increase in the expression of transforming growth factor beta (TGFβ) and also inflammatory and profibrotic markers in glomeruli, which associates with lipotoxicity and insulin resistance. This model has confirmed the key role of PPARγ2 in regulating renal lipid metabolism and insulin sensitivity, which is closely associated with glomerular filtration rate and albuminuria [34]. Furthermore, we showed that podocytes treated with saturated palmitic acid present a tendency for decreased *PPARγ1* expression that correlates with a proinflammatory and proapoptotic state, and with changes in the gene expression of enzymes involved in fatty acid synthesis including a decrease in acyl-CoA carboxylase and fatty acid synthase [36].

In addition to lipid metabolism, PPARγ is involved in adipokine expression from adipose tissue, including adiponectin [37], a circulating plasma protein produced by white adipose tissue that negatively correlates with obesity. Adiponectin stimulates fatty acid oxidation, suppresses hepatic gluconeogenesis, increases insulin sensitivity, and acts to counter the effects of tumor necrosis factor, an inflammatory cytokine [38]. Especially relevant to kidney function, low levels of adiponectin correlate with albuminuria both in mice and humans, and adiponectin is postulated as a renoprotective protein after podocyte injury [39].

Heterozygous mutations in *PPARγ* cause FPLD3 (OMIM 604367), clinically characterized by loss of subcutaneous limb and gluteal fat with preservation of visceral and subcutaneous abdominal fat, fatty infiltration of the liver, and hyperuricemia [14]. All patients develop type II diabetes mellitus and hypertension at an unusually early age. Interestingly, a single case study showed that the adipokine leptin was effective in treating metabolic complications in a patient with FPLD3 [40]. It is known that the kidney expresses a leptin receptor, and that leptin is cleared from the bloodstream principally by the kidney [41].

Systemic lupus erythematosus is an autoimmune connective tissue disease marked by immune complex-mediated lesions in small blood vessels of different organs, particularly the kidneys. Mice lacking macrophage-specific expression of PPARγ or RXRα develop glomerulonephritis and autoantibodies to nuclear antigens, resembling the nephritis seen in systemic lupus erythematosus [42]. Moreover, these mice exhibit tubule-interstitial lipid deposition that leads to lipid-mediated tissue damage in all areas of the nephron [43].

Overall, these findings illustrate the crucial role of PPARγ as a master regulator of systemic lipid metabolism in the kidney.

## 3. PPARγ in Renal Glucose Metabolism

The kidney contributes to glucose homeostasis not only through the processes of utilization (i.e., glucose filtration, reabsorption, and consumption), but it is also increasingly recognized as having a significant role in gluconeogenesis (~20% of all glucose production), and uniquely contributes to plasma glucose regulation by controlling glucose reabsorption from renal tubules following glomerular filtration [44]. Whereas the poorly vascularized, and consequently relatively hypoxic, medulla is a site of considerable glycolysis, the cortex is the renal site of gluconeogenesis. Moreover, the proximal tubule is the only region of the kidney with the appropriate enzymes necessary for gluconeogenesis.

Blood glucose is freely filtered at the glomerulus and is then reabsorbed predominantly in the proximal tubule. This process is performed by two sodium-dependent glucose cotransporter (SGLT) proteins situated in different segments of epithelial cells of the proximal tubule (SGLT2 in the S1 segment and SGLT1 in the S3 segment). Once glucose has been concentrated in the epithelial cells, it diffuses out to the interstitium via specific facilitative glucose transporters (GLUTs) localized at the basolateral membrane [44]. Hyperglycemic conditions lead to a dysfunction of SGLT-mediated glucose transport in proximal tubular cells and promote epithelial–mesenchymal transition (EMT), leading to renal fibrosis. PPARγ agonists have been shown to reverse this hyperglycemia-induced EMT and to restore functional SGLT-mediated glucose uptake [45]. Moreover, PPARγ agonists have significant renoprotective properties in experimental models of nephropathy. Correspondingly, thiazolidinedione compounds (TZDs), a class of insulin-sensitizing drug used in the treatment of type 2 diabetes, and a synthetic PPARγ ligand improve glucose tolerance, which may indirectly ameliorate the progression of chronic kidney disease (CKD). Importantly, it has been suggested that the hypoglycemic action of TZDs relates to the inhibition of gluconeogenesis that is confined to proximal tubule cells, with minimal hepatic consequences [44,46]. For instance, administration of the TZD rosiglitazone to rabbit renal tubules resulted in a ~70% decrease in the rate of gluconeogenesis, accompanied by a ~75% decrease in alanine utilization, and a ~35% increase in lactate synthesis [46]. PPARγ agonists have also been tested in humans, and seem to improve glucose tolerance and reduce the urinary albumin excretion rate, thus indirectly delaying renal disease progression. Unfortunately, two PPARγ agonists, rosiglitazone and pioglitazone, had to be withdrawn from the US and European markets because of cardiovascular disease and bladder cancer as associated side effects [21,47]. Thus, more efforts should be made to discover new PPARγ agonists with beneficial effects on CKD.

A recent review has addressed the association of metabolic traits, specifically glucose metabolism, with PPARγ genetic polymorphisms in humans [48], the most common of which leads to the replacement of alanine for proline at codon 12 (Pro12Ala) in PPARγ2. This polymorphism is associated with a risk of diabetic nephropathy in Caucasians, but no similar association is observed in Asians. Additionally, the Ala-12 polymorphism is associated with a decreased risk of albuminuria [49]. Also, whereas heterozygosity for the frameshift/premature stop codon mutation [A553ΔAAAiT]fs.185[stop186] in the DNA-binding domain of PPARγ is not associated with insulin resistance, individuals doubly heterozygous, with an additional defect in an unrelated gene encoding the muscle-specific regulatory subunit of protein phosphatase 1 (PPP1R3A), exhibit severe insulin resistance [50]. Along this line, Dyment et al. described a woman with biallelic mutations in PPARγ who presented with congenital generalized lipodystrophy, hypertriglyceridemia, hepatosplenomegaly, insulin resistance, and renal failure since birth [51].

The discovery of PPARγ as a target for TZDs prompted the screening of a cohort of subjects with severe insulin resistance for mutations in *PPARγ*. Through this analysis, two new heterozygous mutations in PPARγ, Pro467Leu, and Val290Met, were identified in three subjects [14]. In addition to severe insulin resistance, all three patients developed liver steatosis, type 2 diabetes, and also hypertension at a very early age [52]. Similarly, rare mutations in the ligand-binding domain of PPARγ (Arg425Cys and Phe388Leu) have been found in patients with insulin resistance [53]. Moreover, the novel PPARγ mutations Arg165Thr and Leu339X, which are linked to familial partial lipodystrophies, are associated with a defective transrepression of RAS, leading to cellular dysfunction and contributing to the specific FPLD3-linked severe hypertension [54]. In addition to these findings, other mutations such as Pro115Glu have been identified in nonlipodystrophic subjects, ascertained based upon obesity and diabetes [13].

## 4. PPARγ in Renal Mineral Metabolism

Chronic kidney disease is directly related to the development of abnormalities in bone mineral metabolism. The kidney is known to regulate the levels of vitamin D3 by producing its most active form, 1,25 dihydroxyvitamin D3 (calcitriol), which participates in calcium and phosphate metabolism. The key players in this hormonal bone-parathyroid-kidney axis include fibroblast growth factor 23 (FGF23), Klotho, parathyroid hormone (PTH), and calcitriol, and recent work has also implicated the involvement of PPARγ [55].

Osteoblasts and adipocytes share a common multipotent mesenchymal stem cell progenitor. Akune et al. [56] showed that homozygous *PPARγ*-deficient embryonic stem cells failed to differentiate into adipocytes, but spontaneously differentiated into osteoblasts. They further showed that adipogenesis was restored by reintroduction of the *PPARγ*, indicating that PPARγ insufficiency stimulates osteoblastogenesis in vivo. The canonical Wnt/β-catenin-PPARγ system determines the molecular switch between osteoblastogenesis and adipogenesis [57]. Activation of this pathway leads to osteogenesis, not adipogenesis, and its inhibition leads to an increase in transcription of *PPARγ*. The osteogenic pathway is linked to the stimulation of Wnt signaling, leading to the final transcriptional activation of early osteogenic markers such as runt-related transcription factor 2 (*RUNX-2*) and alkaline phosphatase (*ALP*), which is mediated by β-catenin. Conversely, the adipogenic pathway involves inhibition of the Wnt pathway leading to ubiquitination/degradation of β-catenin, which results in the transcription of *PPARγ*, a pivotal initiator of adipogenesis [58].

It has been recently shown that the murine *klotho* gene is a target of PPARγ [59]. *Klotho* was initially discovered as an antiaging gene and encodes a single-pass transmembrane protein that forms a complex with the FGF receptor (FGFR) to create a de novo high-affinity binding site for FGF23. In addition to membrane-anchored Klotho, a secreted form of the protein is directly released into the extracellular compartment and is present in body fluids [55]. *Klotho* is expressed in multiple tissues but is particularly high in the kidney in distal convoluted tubules, proximal convoluted tubules, and also in inner medullary collecting duct-derived cell lines. Recently, two *Klotho*-related genes were identified based on sequence similarity, *βKlotho* and *γKlotho*. The originally described *Klotho* is now referred to as *αKlotho* to distinguish it from the β and γ forms. *βKlotho* is expressed in various tissues, most notably in the liver and white adipose tissue, whereas *γKlotho* is expressed in the eye [60].

*Klotho* is involved in the regulation of calcitriol production and modulates urinary phosphate, calcium and potassium excretion, and is downregulated in conditions related to kidney injury [61]. Consistent with its activation by PPARγ, oral administration of the PPARγ agonist troglitazone augmented renal *klotho* mRNA expression in Otsuka Long–Evans Tokushima Fatty rats and protected against the endothelial dysfunction induced in this model of atherogenesis [62]. Unfortunately, TZD treatment is associated with an increased risk of hip fractures and is linked to the formation of excessive calcium phosphate precipitates in the urinary bladder [63,64]. Likely, this effect is caused by excess PPARγ activity, which increases adipogenesis and downregulates the β-catenin pathway and osteoblastogenesis. Overall, these data point to PPARγ as an important player in maintaining mineral metabolism, both at the renal and the systemic level.

## 5. PPARγ in Systemic Blood Pressure Control

In line with a major role in the regulation of vascular tone and blood pressure, mutations in *PPARγ* induce severe hypertension and type 2 diabetes. Mice with mutations in *PPARγ* in smooth muscle present vascular dysfunction and severe systolic hypertension [65]. Cells of the juxtaglomerular apparatus express *PPARγ* (Figure 1) and produce renin, a protease that cleaves angiotensinogen to generate angiotensin I. Renin is a key component of the renin-angiotensin-aldosterone system (RAAS) that mediates extracellular volume. The human renin gene is activated by endogenous and pharmacological PPARγ agonists and is a direct target of PPARγ, containing two PPARγ binding sequences that control its transcription [66].

PPARγ also acts as a negative regulator of angiotensin II receptor 1 transcription, another important component of RAAS. In 2004, the angiotensin II receptor 1 antagonist telmisartan, which is to treat hypertension and diabetes, was identified as a partial agonist of PPARγ [67,68]. By activating PPARγ, telmisartan exerts beneficial effects on the kidney by decreasing proteinuria and inflammation, and consequently confers renoprotection. Since telmisartan can bind to the ligand-binding domain of PPARγ at a site that is different to that used by TZDs, it is not surprising that telmisartan possesses unique properties unrelated to that of conventional TZDs. Accordingly, telmisartan has the capacity to reverse the progression of EMT induced by TGFβ1 in cultured human kidney proximal tubule epithelial cells and can counteract EMT-related pathological changes such as renal fibrosis [69]. The combined use of TZDs and angiotensin receptor blockers, however, fails to provide synergistic protective action, and it has been shown that co-administration of RAAS inhibitors and PPARγ agonists promotes anemia in uncomplicated diabetic patients [70]. The development of new therapies (combined or not) that exploit the beneficial effects of PPARγ activation in the treatment of renal disease are therefore warranted.

It has been also observed that troglitazone has vasodilating effects on efferent and afferent arterioles from rabbit kidney, consistent with PPARγ expression in intima/media renal vasculature, thereby decreasing glomerular capillary pressure and hence excretion of urinary protein [71].

**Figure 2:**
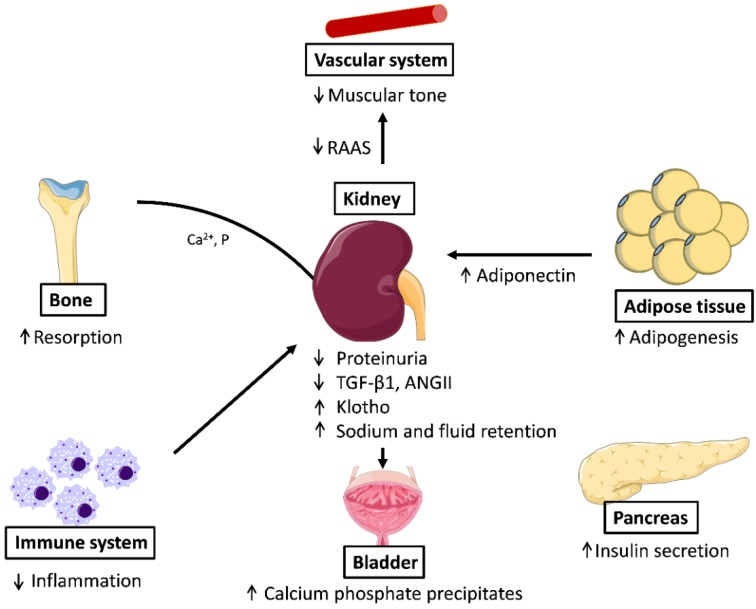
Effects of PPARγ activation in different systems: In the vascular system, PPARγ decreases arterial pressure, relaxes muscular tone, and downregulates RAAS; in adipose tissue, the activation of PPARγ promotes adipogenesis; in β-cells it increases insulin secretion; PPARγ is also anti-inflammatory and from the bone resorption is produced accompanied of release of calcium and phosphate that form stones in the bladder. ANG-II: Angiotensin II; RAAS, Renin Angiotensin Aldosterone System; TGF-β1, transforming growth factor beta 1.

The beneficial effects of PPARγ agonists in the control of blood pressure underscore the pivotal role of PPARγ at this level. PPARγ also has a critical role in systemic fluid retention through the regulation of renal sodium transport in the collecting duct, as the adverse effect of TZD use in the treatment of diabetes is PPARγ-dependent. Thus, the *Scnn1g* gene, encoding the gamma subunit of the epithelial Na^+^ channel, was identified as a critical PPARγ target gene in the control of edema [72,73]. The TZD-induced fluid retention effects are attenuated in patients by the combination treatment with diuretics (Figure 2).

## 6. PPARγ and Circadian Rhythm

All PPAR isoforms are known to be rhythmically expressed [25]. Specifically, PPARγ has direct interactions with the core clock genes [26,27,28], suggesting that it may act as a molecular link between circadian rhythm and energy metabolism. Moreover, PPARγ exhibits variations in its diurnal expression in mouse fat, liver, and blood vessels [74], which is exacerbated by consumption of a high-fat diet [75]. In addition, deletion of *PPARγ* in mouse suppresses or diminishes diurnal rhythms [76]. In this regard, *nocturnin*, a circadian-regulated gene that encodes a deadenylase thought to be involved in the removal of polyA tail from mRNAs, binds to PPARγ and enhances its transcriptional activity. Enhanced activity of PPARγ by nocturnin may result in increased bone marrow adiposity and bone loss [77].

The circadian expression of PPARγ has not yet been established in kidney. An evaluation of this phenomenon could be especially interesting to associate the regulation of renal metabolism with central and peripheral circadian networks.

## 7. Conclusions

PPARγ is a nuclear receptor that regulates systemic glucose and lipid homeostasis and participates also in immunity and vascular health. In the kidney, PPARγ is expressed in many different types of cells with diverse metabolic specializations, underscoring important roles for this nuclear receptor in renal lipid metabolism, glucose management, mineral metabolism, and the control of systemic blood pressure. PPARγ activation improves insulin sensitivity and reduces cardiovascular complications and renal injury in clinical practice. Nevertheless, the complex regulation of *PPARγ* together with the adverse effects of PPARγ agonists hinders efforts to develop safe clinical treatments. The new challenges with regard to future applications include a comprehensive analysis of PPARγ cell-specific actions in the kidney and the manipulation of PPARγ expression. Finally, a better understanding of the molecular mechanisms of action of PPARγ in specific pathways together with its systemic implications may allow the development of new agonists and modulators to improve the management of kidney disease and consequently global homeostasis.

## Abbreviations

CKD	chronic kidney disease
EMT	epithelial-mesenchymal transition
FFA	free fatty acids
FGF23	fibroblast growth factor 23
GLUT	glucose transporter
PPAR	Peroxisome proliferator-activated receptors
PPRE	PPAR response element
RAAS	Renin Angiotensin Aldosterone System
RXR	retinoid X receptors
SGLT	sodium-dependent glucose cotransporter
TGFβ	transforming growth factor beta
TZD	thiazolidinedione
